# QuickStats

**Published:** 2013-05-03

**Authors:** Debra Blackwell, Tainya C. Clarke

**Figure:**
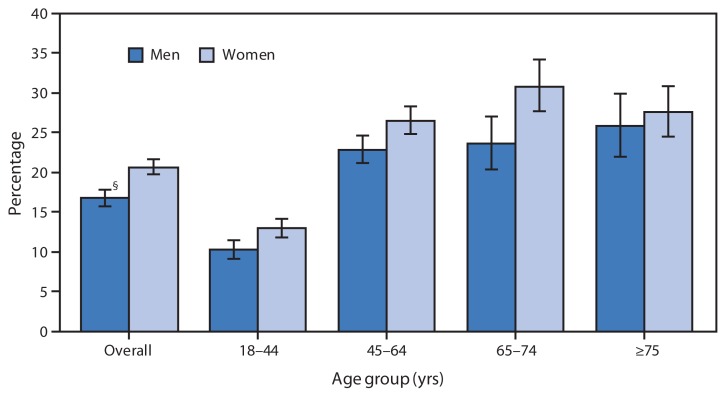
Percentage of Adults Aged ≥18 Years Who Often Had Pain in the Past 3 Months,^*^ by Sex and Age Group — National Health Interview Survey, United States, 2010–2011^†^ ^*^ Based on responses to the following questions: “In the past 3 months, how often did you have pain? Would you say never, some days, most days, or every day?” Persons who had pain most days or every day were categorized as often having pain. Unknowns were not included in the denominators when calculating percentages. ^†^ Estimates are based on household interviews of a sample of the noninstitutionalized U.S. civilian population. Estimates are age-adjusted using the projected 2000 U.S. population as the standard population and using four age groups: 18–44 years, 45–64 years, 65–74 years, and ≥75 years. ^§^ 95% confidence interval.

During 2010–2011, women (20.7%) were more likely than men (16.9%) to often have pain overall and in all age groups except those aged ≥75 years. Among both men and women, those aged 18–44 years were less likely to often have pain than adults in older age groups.

**Source:** National Health Interview Survey, 2010 Quality of Life and 2011 Functioning and Disability supplements. Data are from a subset of the adults randomly selected for the Sample Adult Component of the National Health Interview Survey questionnaire. Available at http://www.cdc.gov/nchs/nhis.htm.

